# Assessment of leachables and extractables in “super-swelling” hydrogel-forming microarray patches

**DOI:** 10.1007/s13346-025-01880-2

**Published:** 2025-05-31

**Authors:** Qonita Kurnia Anjani, Peter E. McKenna, Eneko Larrañeta, Panagiotis Manesiotis, Yidan Luo, Masoud Adhami, Fabiana Volpe-Zanutto, Gareth Orr, Sabrina Roussel, Ryan F. Donnelly

**Affiliations:** 1 School of Pharmacy, Medical Biology Centre, Queen’s University Belfast, 97 Lisburn Road, Belfast, BT9 7BL UK; 2 School of Chemistry and Chemical Engineering, David Keir Building, 39-123 Stranmillis Road, Belfast, BT9 5 AG UK; 3 School of Biomedical Sciences, Mass Spectrometry Centre, Ulster University, Cromore Road, Coleraine, BT52 1SA UK

**Keywords:** Leachables, Extractables, Hydrogel-forming microarray patches, Microneedles, Transdermal

## Abstract

Hydrogel-forming microarray patches (MAPs) offer a minimally invasive platform for transdermal drug delivery, enabling systemic absorption of active pharmaceutical ingredients. Unlike dissolving MAPs, which deposit their entire polymer matrix into the skin, hydrogel-forming MAPs remain intact upon removal, reducing polymer exposure while delivering higher drug doses than dissolving or coated MAPs. Moreover, they have demonstrated excellent biocompatibility and do not cause skin or systemic issues, even with repeated application in humans. This study assessed the leachable and extractable compounds from hydrogel-forming MAPs composed of Gantrez^®^ S-97, PEG 10,000, and sodium carbonate under various conditions. Under physiological conditions (37°C in water), minimal PEG 10,000 leaching (10.4 ± 2.0%) and negligible Gantrez^®^ S-97 extraction (< 2%) confirmed the hydrogel matrix’s stability and safety. However, stress testing in DMSO at 70°C led to increased PEG 10,000 extraction (up to 32.9 ± 6.1%) and minor Gantrez^®^ S-97 degradation, likely due to ester hydrolysis. These findings highlight the robustness of hydrogel-forming MAPs, ensuring minimal systemic exposure to unbound polymers while maintaining effective drug delivery. The results support their potential for chronic therapeutic applications requiring repeated dosing. Further clinical studies are needed to validate these findings, facilitating regulatory approval and broader adoption across diverse medical applications.

**Graphical Abstract:**

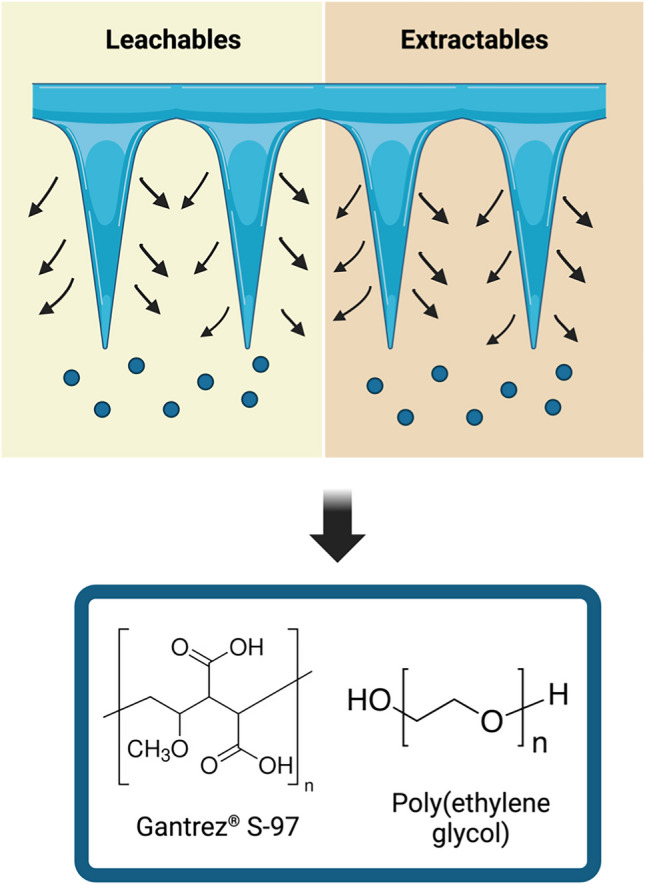

## Introduction

Hydrogel-forming microarray patches (MAPs) represent an advanced transdermal drug delivery platform, providing a minimally invasive method for systemic absorption of active pharmaceutical ingredients. These MAPs are produced through a crosslinking process between hydrophilic polymers and a crosslinking agent, forming an insoluble yet swellable polymer network due to ester bond formation during crosslinking [1]. Upon application to the skin, they absorb interstitial fluid, causing the hydrogel matrix to swell, which facilitates drug diffusion from an attached reservoir layer into the dermal microcirculation, ultimately achieving systemic absorption [2]. Unlike other MAP types, hydrogel-forming MAPs do not contain active compounds within the needle structure [3, 4], instead, drugs are stored in a separate reservoir positioned on top of the patch. This design enables the delivery of high doses of a wide range of compounds [1, 3, 5–12]. Hydrogel-forming MAPs remain macroscopically intact even after five days of application [13, 14]. Previous studies have demonstrated their excellent biocompatibility [5], with no reported skin irritation or systemic adverse effects, even after repeated applications in humans [15].

We have previously demonstrated that Gantrez^®^ S-97, crosslinked with PEG 10,000, possesses the capability, in terms of mechanical strength, to pierce the *stratum corneum* [16]. The term'super swelling'was used due to the addition of anhydrous sodium carbonate (Na_2_CO_3_) as a modifying agent, which reduces the ester-based crosslinking through the formation of sodium salt with the free acid groups of Gantrez^®^ S-97 [16]. The ability of super swelling hydrogel-forming MAPs to deliver a range of drugs with diverse physicochemical properties has been reported [3, 7, 16–18]. Furthermore, our extensive studies have demonstrated that hydrogel-forming MAPs are biocompatible, possess intrinsic antimicrobial characteristics, and inhibit microbial proliferation [2, 19, 20]. Repeat application of these MAPs to the skin of human volunteers, followed by a 24-h retention period, did not result in any adverse effects on skin barrier function, skin appearance, or systemic indicators of infection, immunity, inflammation, or allergies [21]. We have also shown that human volunteers can consistently insert these MAPs into their own skin, even when the patch size exceeds the conventional range of 1–2 cm^2^ normally observed in microneedle systems [20, 22, 23].

Given the performance of hydrogel-forming MAPs, which absorb interstitial fluid before facilitating drug permeation from the reservoir into deeper skin layers, it is crucial to assess the compounds released during the swelling process. While the materials used in the fabrication of hydrogel-forming MAPs, such as poly(ethylene glycol) (PEG) and Gantrez^®^ S-97, are components of approved drugs and medical products, respectively, there remains a possibility that polymers released from the system could enter systemic circulation and be primarily excreted via the kidneys. However, it is widely recognised that glomerular filtration retains substances with a high molecular weight (cutoff 30–50 kDa) [24]. Given that Gantrez^®^ S-97 has a molecular weight of 1,200 kDa, its potential filtration by the glomerulus warrants careful consideration. Therefore, determining leachable and extractable compounds is essential for the translation of this technology, a focus addressed for the first time in this study. Extractables are defined as organic or inorganic chemical entities that can be released from a material under exaggerated conditions, such as strong solvents, elevated temperatures, or prolonged exposure, and are used to simulate worst-case scenarios [25, 26]. In contrast, leachables are compounds that migrate from the material under normal use conditions, such as during application to the skin or contact with biological tissues, and therefore represent actual patient exposure [25–27]. Identifying and quantifying potential leachables, compounds that may migrate into the patient during normal use, provides critical safety insights for hydrogel-forming MAPs [28, 29].

## Materials and methods

### Materials

Gantrez^®^ S-97, with a molecular weight of 1,200 kDa is a copolymer of methylvinylether and maleic acid (PMVE/MA) and was supplied by Ashland (Kidderminster, UK). Phosphate buffered saline (PBS) tablets with a pH of 7.4, polyethylene glycol (PEG) with a molecular weight of 10 kDa, dimethyl sulfoxide (DMSO) and sodium bicarbonate were purchased from Sigma-Aldrich (Dorset, UK). A water purifying system produced ultrapure water (Elga PURELAB DV 25, Veolia Water Systems, Dublin, Ireland).

### Preparation of hydrogel-forming MAPs

Hydrogel-forming MAPs were prepared using an aqueous blend consisting of 20% w/w Gantrez^®^ S-97, 7.5% w/w PEG, and 3% w/w Na₂CO₃, following a previously established method [1, 3]. To eliminate air bubbles, the blend was centrifuged at 3,500 rpm for 15 min using an Eppendorf^®^ 5804 centrifuge (Fisher Scientific, Loughborough, UK). Pre-formed silicone microneedle moulds (11 × 11 array; conical shape; 700 µm needle height; 300 µm base width; 300 µm interspacing) were filled with 500 mg of the blend and centrifuged again under the same conditions to ensure complete mould cavity filling.

The silicone moulds were fabricated using a custom mould holder lined with poly(dimethyl siloxane) (PDMS), containing female moulds created via laser engineering (Blueacre Technology, Dundalk, Ireland). The master mould used to produce the silicone moulds was designed in Tinkercad^®^ software and printed using an Ultimaker Cura 4.4 3D printer with polypropylene filament. The printed master mould had an overall diameter of 3 cm and a thickness of 2 cm, with a central square section measuring 1.2 cm in height, 1.2 cm in width, and 1 cm in thickness. To prepare the silicone moulds, 5 g of silicone elastomer base and curing agent were mixed in a 10:1 ratio, centrifuged at 2,500 rpm for 10 min to remove air bubbles, and then slowly poured into the printed master mould. The mixture was cured at room temperature for 24 h, after which the moulds were carefully removed using a metallic spatula.

The filled moulds were then left to dry at room temperature for 48 h. After drying, the sidewalls of the patches were trimmed, and the MAPs were crosslinked in an oven at 80 °C for 24 h. The final MAPs measured approximately 0.6 cm × 0.6 cm, with individual microneedles measuring 700 µm in height, 300 µm in base width, and spaced 300 µm apart.

### Experimental set up

**Fig. 1:**
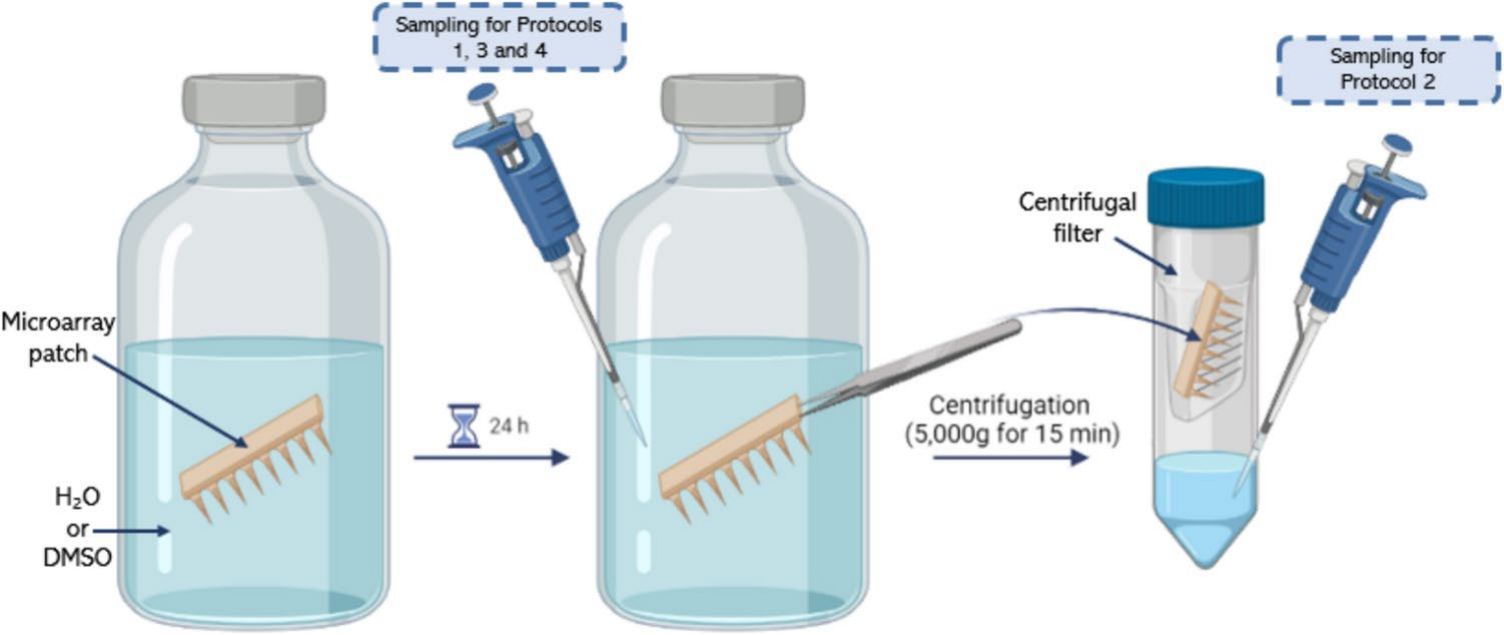
Schematic representation of the experimental set-up implemented during determination of leachables and extractables

To identify leachable and extractable compounds from hydrogel-forming MAPs, protocols were developed following guidance from the US and European Pharmacopoeias, the FDA (United States Food and Drug Administration), and the EMA (European Medicines Agency) [30–35]. Briefly, individual hydrogel-forming MAPs (119.7 ± 2.8 mg) were placed in 20 mL of either H₂O or dimethyl sulfoxide (DMSO) and incubated for 24 h, as outlined in Table 1. For protocols one, three, and four, 1 mL samples of the unabsorbed H₂O/DMSO remaining in the vial were collected for analysis. In protocol two, each swollen hydrogel MAP was transferred to an Amicon^®^ Ultra-4 centrifugal filter unit (100 kDa molecular weight cut-off) and centrifuged at 3,500 rpm for 15 min to extract absorbed H₂O. A 1 mL sample of the extracted H₂O was then collected for analysis (Fig. 1).

**Table 1:** Details of protocols implemented during leachables and extractables experimentation

Protocol No.	Incubation temperature (℃)	Sample taken from	Test type
1	37	Remaining H_2_O	Leachable
2	37	H_2_O extracted from swollen MAP	Extractable
3	70	Remaining DMSO	Extractable
4	90	Remaining H_2_O	Extractable

### Moisture content assessment

To determine the proportions of each material (in mg) in the MAPs, moisture content was assessed to quantify the water content in each MAP, allowing for the theoretical calculation of PEG and Gantrez^®^ S-97 content. Moisture content was measured using a Kern DBS 60–3 moisture analyser (Kern & Sohn GmbH, Balingen-Frommern, Germany). The analysis was performed in “slow” mode with the heating temperature set at 150 °C. The measurement process lasted approximately six minutes.

### Liquid chromatography-mass spectrometry (LC–MS) analysis

To identify polymeric compounds related to PEG 10,000 in all tested samples, liquid chromatography–mass spectrometry (LC–MS) analysis was performed using an Agilent 1290 Infinity UPLC system equipped with a 6125B mass selective detector (MSD). Separation was achieved using a Zorbax SB-C18 column (2.1 × 50 mm, 1.8 μm) maintained at 30 °C. A gradient elution was applied, starting from 90% water and 10% methanol (both containing 0.1% formic acid) to 100% methanol with 0.1% formic acid over 8 min, at a flow rate of 0.2 mL/min. The mass spectrometer was operated in positive ion mode, scanning over an m/z range of 100–2,000 amu.

### HPLC-evaporative light scattering detector (HPLC-ELSD) analysis

To analyse and quantify PEG 10,000 released from samples collected under different experimental conditions, an Agilent 1260 Infinity HPLC system equipped with an evaporative light scattering detector (ELSD) was used. Separation was achieved using a Phenomenex Kinetex™ C18 column (4.6 × 150 mm, 5 μm) maintained at 30 °C. A gradient elution was applied, transitioning from 90% water and 10% acetonitrile to 100% acetonitrile over 7 min, at a flow rate of 1 mL/min. The ELSD was operated with the evaporator and nebuliser temperatures set at 50 °C and a nitrogen (N₂) gas flow rate of 1.60 SLM. Quantitative results were compared against the theoretical PEG content, which was calculated based on the weight of MAPs after crosslinking for 24 h and removal of the sidewalls.

### FTIR analysis

In this study, Fourier transform infrared (FTIR) spectroscopy was used to analyse the chemical composition of samples obtained from the leachable and extractable studies. Liquid samples were first frozen at − 80 °C for 3 h and subsequently lyophilised using a freeze dryer (Virtis™ Advantage XL-70, SP Scientific, Warminster, PA, USA) for 24 h to eliminate residual moisture and avoid liquid-phase interference. The lyophilisation cycle consisted of primary drying at − 40 °C and secondary drying at 25 °C, under a pressure of 50 mTorr, as described previously [3].

FTIR analysis was conducted using an Accutrac FT/IR-4100 Series spectrometer (Jasco, Essex, UK) equipped with a MIRacle diamond attenuated total reflectance (ATR) accessory (Pike Technologies Ltd., Madison, WI, USA). Spectra were collected at room temperature in the range of 4000–600 cm⁻^1^, with a resolution of 4.0 cm⁻^1^. Each spectrum was averaged over 32 scans to improve signal quality and measurement accuracy.

### NMR analysis

The dried powder obtained from each study sample was subjected to lyophilisation and subsequently analysed using proton nuclear magnetic resonance (^1^H-NMR) spectroscopy to determine the Gantrez^®^ S-97 content. Samples (after lyophilisation) were dissolved in deuterated dimethyl sulfoxide (DMSO-d₆), and spectra were acquired using a Bruker Ultrashield™ 400 spectrometer (Bruker, Leipzig, Germany) operating at 400 MHz. Spectral data were processed using MestReNova 6.0.2© software (Mestrelab Research, Santiago de Compostela, Spain). Chemical shifts are reported in parts per million (ppm, δ), referenced to tetramethylsilane or the residual solvent peak. 1H-NMR was used to quantify the amount of Gantrez^®^ S-97 leached from the MAPs by comparing the integration areas of characteristic proton peaks assigned to PEG and Gantrez^®^ S-97. The number of protons contributing to each peak and the amount of PEG leached, previously quantified using HPLC-ELSD, were used to calculate the corresponding Gantrez^®^ S-97 content. This calculation was performed using **Eq. **1.

1 $${m}_{Gan}= \frac{{A}_{Gan}}{{A}_{PEG}}\cdot {m}_{PEG}\cdot \frac{{Mw}_{Gan}}{{Mw}_{PEG}}\cdot \frac{{nH}_{Gan}}{{nH}_{PEG}}$$

where m_Gan_ is the amount of Gantrez^®^S-97 leached from the samples, A_Gan_ is the area of the NMR signal peak assigned to Gantrez^®^ S-97 protons, A_PEG_ is the area of the NMR signal assigned to PEG protons, m_PEG_ is the mass of PEG leached from the samples that was measured using HPLC-ELSD, Mw_Gan_ is the molecular weight of Gantrez^®^ S-97 (1,200 kDa), Mw_PEG_ is the molecular weight of PEG (10 kDa), nH_Gan_ is the number of protons assigned to the Gantrez^®^ S-97 peak and nH_PEG_ is the number of protons assigned to the PEG peak.

### MALDI-TOF analysis

To evaluate the presence of PEG released from the samples, matrix-assisted laser desorption/ionisation time-of-flight mass spectrometry (MALDI-TOF MS) was performed. Analyses were conducted using an AB SCIEX 4800 Plus MALDI TOF/TOF™ Analyzer operating in linear mid-mass positive mode, with a mass scan range of 2,000–20,000 Da. The laser intensity was set to 4,800, and 500 laser shots were accumulated per spectrum. Data processing was carried out using AB SCIEX Data Explorer^®^ Software, applying baseline correction and Gaussian noise smoothing (filter width: 5 points).

For sample preparation, 10 µL of each sample was mixed with 10 µL of α-cyano-4-hydroxycinnamic acid (CHCA) matrix, prepared at 5 mg/mL in an 80:20 (v/v) acetonitrile:water solution containing 0.1% trifluoroacetic acid. The mixture was vortexed at 1,500 rpm for 1 min. A 0.5 µL aliquot of the resulting solution was then spotted onto a 384-well Opti-TOF MALDI plate pre-spotted with an external calibrant solution (PepMix IV, Bruker Daltonics).

## Results

### Preparation of hydrogel-forming MAPs

**Fig. 2:**
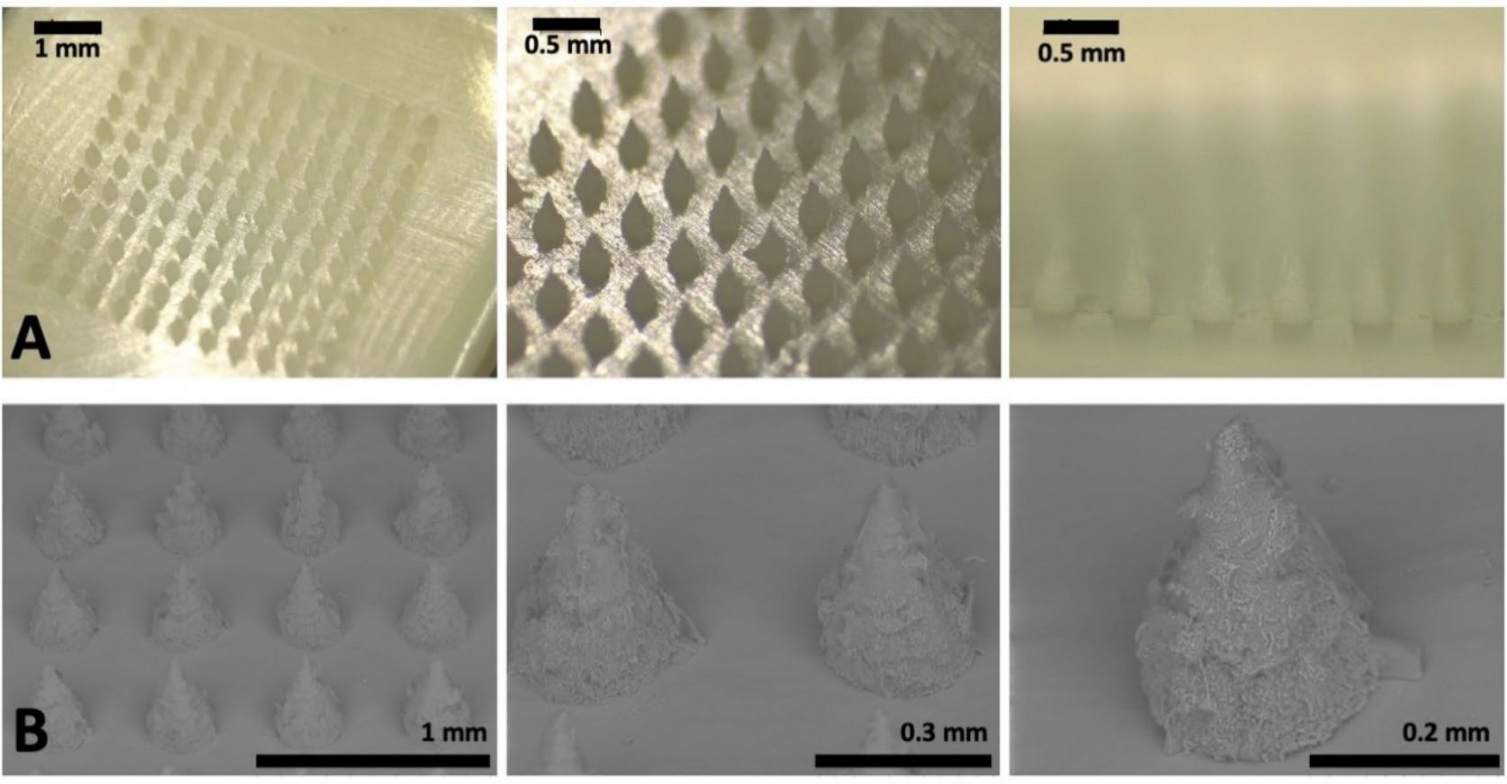
Representative (**A**) digital and (**B**) SEM images of “super-swelling” hydrogel-forming MAP

The digital and SEM images of the “super-swelling” hydrogel-forming MAPs are presented in Fig. 2. All prepared MAPs displayed a homogeneous polymer distribution, with clearly defined, sharp needle tips measuring 572.2 ± 26.1 µm in height. The needles had a conical shape with rough surface, resulting from the laser-engineered PDMS moulds used in this study. These MAPs were subsequently placed under different conditions to evaluate leachables and extractables.

### LC–MS analysis

**Fig. 3:**
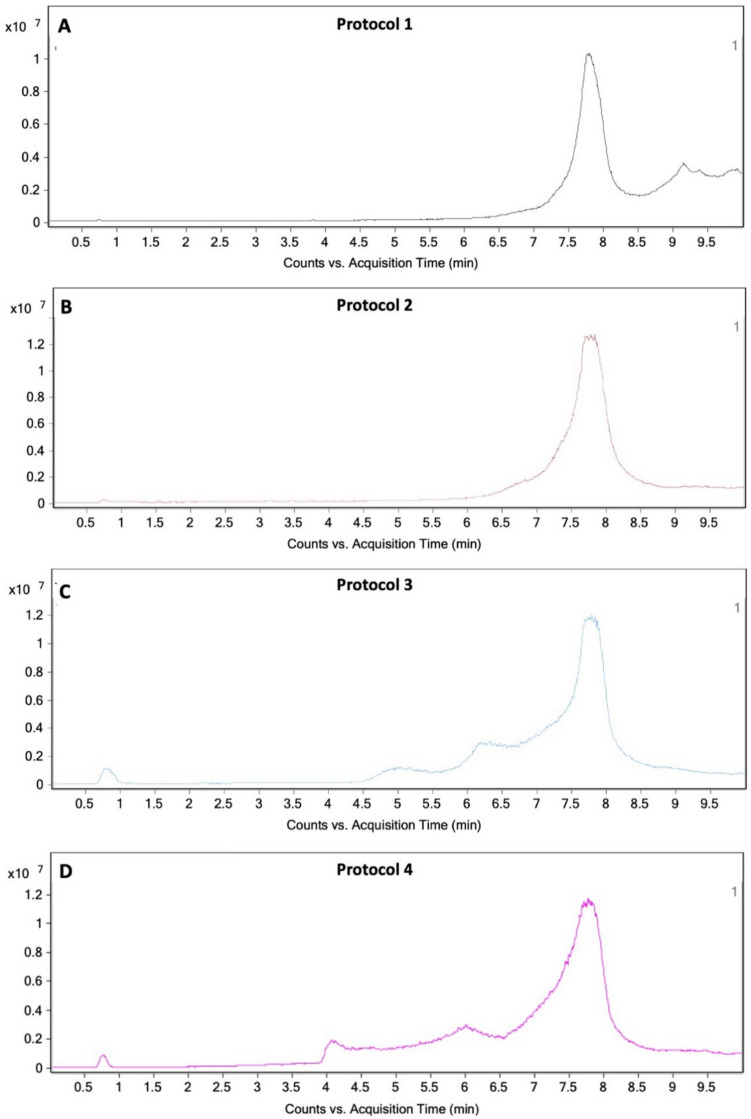
Total ion chromatograms (TIC) of samples obtaining from (**A**) Protocol 1, (**B**) Protocol 2, (**C**) Protocol 3 and (**D**) Protocol 4 showing the retention time and intensity of detected compounds. The prominent peak at ~ 7.7 min corresponds to PEG 10,000 confirming its presence in all samples. These data were acquired in TIC mode for qualitative analysis

LC–MS analysis identified polymeric compounds related to PEG 10,000 in all tested samples, with variations in relative abundance and distribution across different protocols. The analysis was conducted in total ion chromatogram (TIC) mode to qualitatively confirm the presence of PEG 10,000. As shown in Fig. 3, the chromatograms illustrate the elution profiles of the sample components, with a peak at approximately 7.7 min corresponding to PEG 10,000. While TIC mode does not allow for reliable quantification, relative comparisons of peak areas suggest that Protocols 2 and 4 exhibited higher levels of polymeric compounds than Protocols 1 and 3, with Protocol 4 showing the greatest abundance.

This trend may be attributed to the high temperature (90 °C) used in Protocol 4, which likely promoted ester hydrolysis in the swollen hydrogel-forming MAPs [36]. Additionally, incomplete crosslinking during the manufacturing process may have contributed to the release of uncrosslinked PEG. Gel fraction analysis conducted in water at 37 °C and 90 °C revealed retention rates of approximately 88.62 ± 1.62% and 79.20 ± 9.74%, respectively, suggesting that some free PEG dissolved into the medium during incubation. These results indicate that processing conditions in Protocols 2 and 4 may enhance the release or extractability of polymeric materials. Although PEG-related compounds were detected by LC–MS, precise identification of individual species would require further analysis using reference standards. No additional compounds were detected under the current experimental conditions, confirming the predominance of PEG-derived polymers across all protocols.

### HPLC-ELSD analysis

**Fig. 4:**
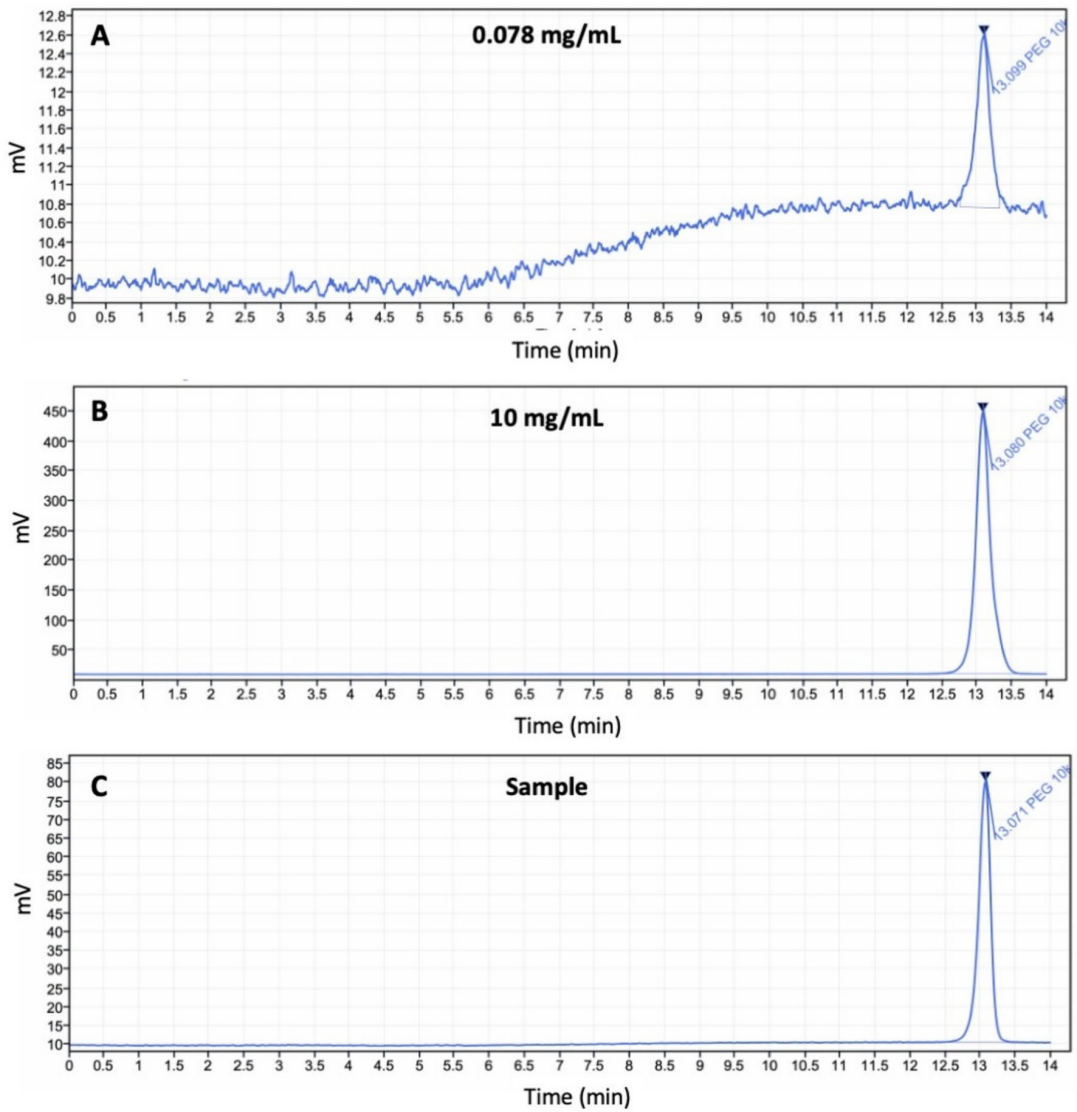
Representative chromatograms of PEG 10,000 Da at (**A**) the lowest concentration (0.078 mg/mL), (**B**) the highest concentration (10 mg/mL), and (**C**) a sample obtained following the study protocol

The quantification results from HPLC-ELSD analysis align with the LC–MS findings, as shown in Table 2, indicating varying levels of extracted PEG across different protocols. The representative chromatograms are shown in Fig. 4. The theoretical PEG content was calculated after calculating the dry mass of MAPs. To determine the proportion of each component in the hydrogel-forming MAPs, the experimentally measured moisture content was used to calculate the residual water and solid content. The total solids in the initial aqueous blend were 30.5% w/w, comprising 20% Gantrez^®^ S-97, 7.5% PEG, and 3% Na₂CO₃. Based on the measured residual moisture content of 8.14 ± 0.99%, the remaining 91.86% was attributed to the solid components. The proportion of each material in the final MAP was then estimated by scaling the original solid content relative to this dry fraction. Accordingly, the final composition of the MAPs was approximately 60.26% Gantrez^®^ S-97, 22.60% PEG, 9.04% Na₂CO₃, and 8.14% residual water. Using the starting mass of the hydrogel-forming MAPs and the volume of water remaining after 24 h, the total amount of PEG 10,000 leached from the MAPs was calculated, as detailed in Table 2.

**Table 2:** Poly(ethylene glycol) content leached/extracted from hydrogel-forming MAPs (means ± S.D., n = 3)

Protocol	Starting mass of MAPs	Theoretical PEG content (mg)	Amount of PEG leached/extracted (mg)	Percentage PEG leached/extracted (%)
1	118.9 ± 2.2	26.9 ± 0.5	2.8 ± 0.5	10.4 ± 2.0
2	119.8 ± 3.0	27.1 ± 0.7	4.0 ± 1.4	14.8 ± 4.6
3	120.4 ± 3.6	27.2 ± 0.9	5.3 ± 2.6	19.5 ± 8.6
4	118.5 ± 1.5	26.8 ± 0.4	8.8 ± 1.9	32.9 ± 6.1

**Fig. 5:**
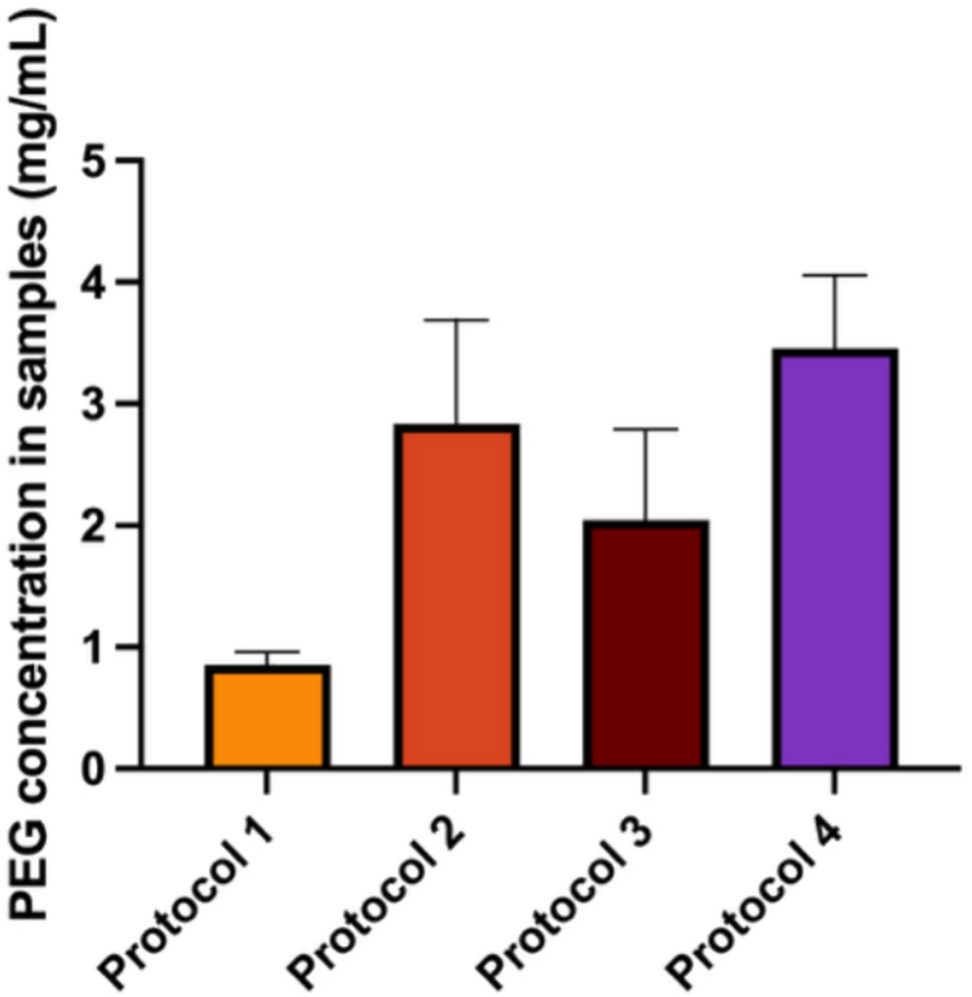
PEG concentration (mg/mL) extracted from each study protocol (Protocol 1 to 4), presented as means ± SD (n = 3)

As shown in Fig. 5, Protocol 1 exhibited the lowest level of PEG extraction, with a mean concentration of 0.86 ± 0.15 mg/mL. Protocol 2 resulted in a higher extraction level, with a mean of 2.84 ± 1.48 mg/mL. Protocol 3 showed intermediate levels, greater than Protocol 1 but lower than Protocol 2, with a mean of 2.05 ± 1.29 mg/mL. Protocol 4 demonstrated the highest extraction, with a mean of 3.46 ± 1.04 mg/mL. These results suggest that elevated temperatures may promote ester hydrolysis, as previously reported [36]. The findings further validate the LC–MS results, emphasising the influence of processing conditions on compound extraction. However, it is important to note that during handling and application, MAPs will not be subjected to the extreme conditions used in Protocols 2, 3, and 4. Since MAPs are crosslinked at 80 °C for 24 h during preparation, prolonged exposure to temperatures exceeding this threshold could accelerate ester hydrolysis, leading to PEG dissolution into the surrounding medium.

### FTIR analysis

**Fig. 6:**
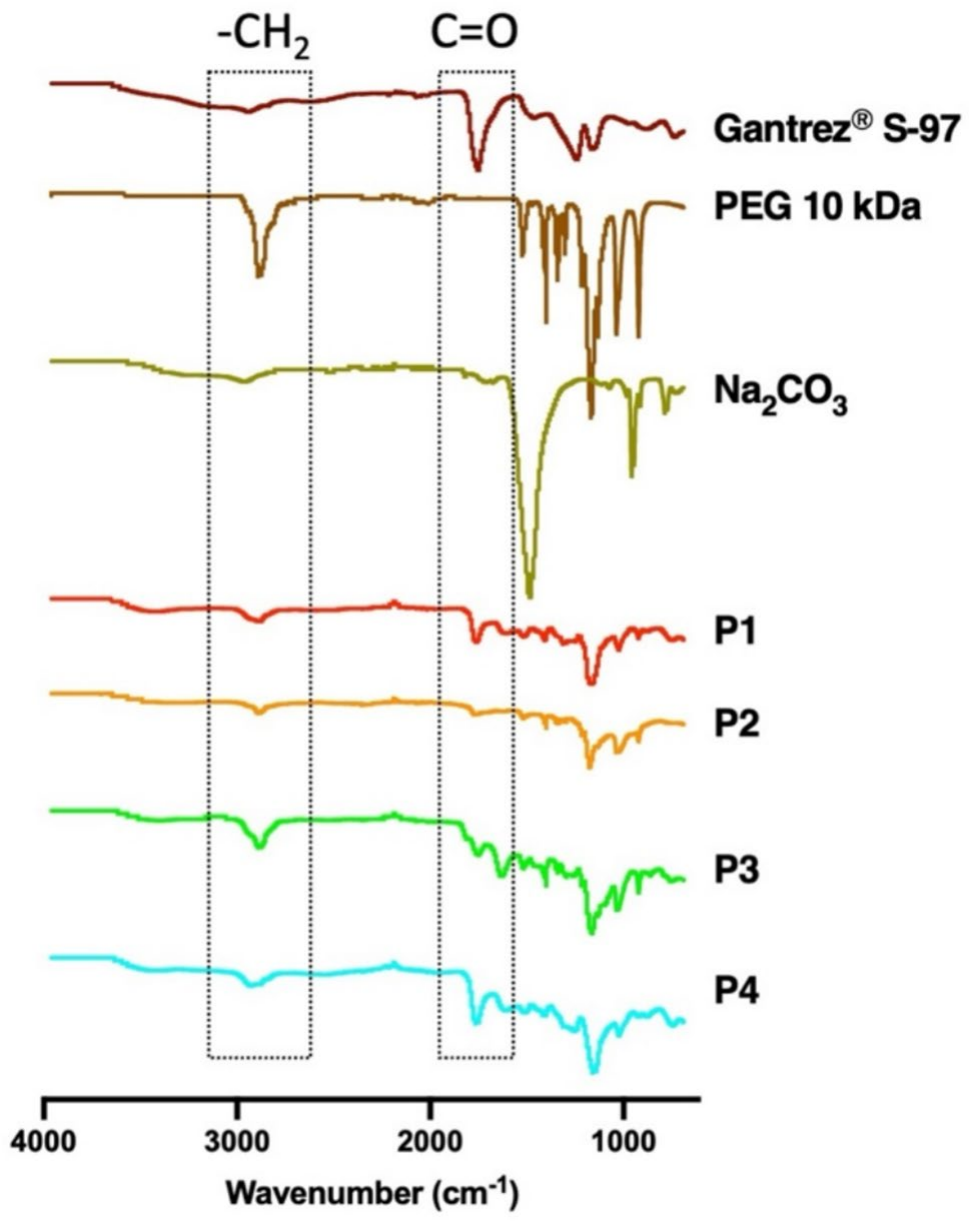
FTIR spectra of the dried samples obtained from Protocol 1, 2, 3, and 4 compared to the pure materials of hydrogel-forming MAPs, Gantrez^®^ S-97, PEG, and Na_2_CO_3_

In this study, FTIR spectroscopy was employed to analyse the chemical composition of samples obtained from the experimental setup. The results shown in Fig. 6. The CH_2_ stretching in the range of 2700–2500 cm⁻^1^ and the carboxylic acid (C = O) band in the range of 1750–1700 cm⁻^1^ were selected as representative markers for PEG and Gantrez^®^ S-97 [37, 38], respectively. Protocol 1 samples exhibited spectral signatures indicative of the presence of both PEG and Gantrez^®^ S-97. In contrast, the spectra for Protocols 2 and 3 were dominated by bands characteristic of PEG, identifying it as the primary polymeric component. Similarly, the spectra for Protocol 4 suggested the presence of both PEG and Gantrez^®^ S-97, consistent with the formulation composition. These findings emphasise the variability in chemical constituents across the different protocols and underscore the influence of experimental conditions on the leached and extracted components.

### NMR analysis

**Fig. 7:**
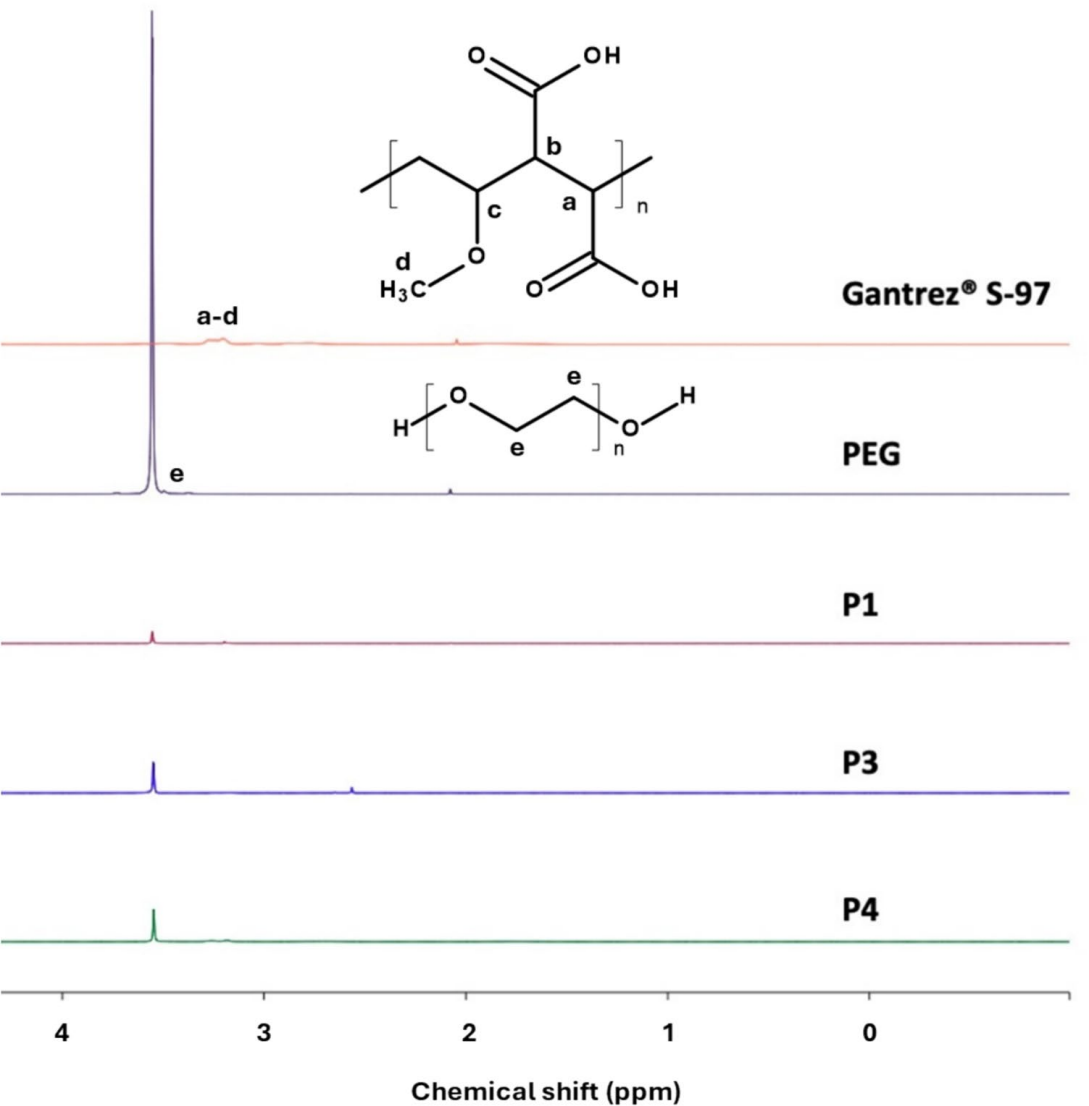
Representative NMR spectra for Gantrez^®^ S-97, PEG and the P1, P3 and P4 extracts

Figure 7 shows the NMR spectra of pure Gantrez^®^ S-97 and PEG. To calculate the amount of Gantrez^®^ S-97 leaching from the MAP, as described in the protocols outlined earlier, we used Eq. 1. For this calculation, we utilised the PEG peak at approximately 3.6 ppm, which corresponds to four protons from two CH_2_ groups (Fig. 7). In contrast, the NMR spectra of Gantrez^®^ S-97 display peaks between 3.0 and 3.5 ppm. These peaks have been attributed to the protons highlighted in Fig. 7, as previously described in the literature [39]. Using the areas of these peaks, the amount of PEG leached for each protocol, the molecular weight of each polymer, and the number of protons assigned to each signal, Eq. 1 was used determine the amount of Gantrez^®^ S-97 leached from the samples. It is important to note that the number of protons assigned to each signal was calculated by multiplying the number of protons in the monomer by the number of repeating units in each polymer. Additionally, after calculating the molar ratio of Gantrez^®^ S-97 and PEG reacting, and assuming complete drying, each -OH group of PEG is associated with more than 153 -COOH groups of Gantrez^®^ S-97.

Table 3 presents the amount of Gantrez^®^ S-97 leached under each protocol. Lower quantities of Gantrez^®^ S-97 were extracted from the MAPs compared to PEG. When the leachables are expressed as a percentage of the initial loading, it is evident that less than 2% of the initial Gantrez^®^ S-97 is extracted using protocol 1. Employing an organic solvent such as DMSO to maximise polymer extraction yields amounts of Gantrez^®^ S-97 comparable to those extracted with water. These findings suggest that less than 2% of the initial Gantrez^®^ S-97 is not bonded to the hydrogel structure.

**Table 3:** Gantrez^®^ S-97 content extracted from hydrogel-forming MAPs (means ± S.D., n = 3)

Protocol	Starting mass of MAPs	Theoretical Gantrez^®^ S-97 content (mg)	Amount of Gantrez^®^ S-97 extracted (mg)	Percentage Gantrez^®^ S-97 leached/extracted (%)
1	106.3 ± 5.8	64.0 ± 3.8	1.2 ± 0.3	1.9 ± 0.5
3	104.3 ± 2.8	62.8 ± 1.8	0.8 ± 0.2	1.3 ± 0.3
4	110.2 ± 7.6	66.3 ± 5.0	2.4 ± 1.1	3.6 ± 0.4

When higher temperatures are used, the amount of Gantrez^®^S-97 extracted increases to approximately 3.3% of the initial loading, likely due to ester hydrolysis as previously reported [36]. Interestingly, the quantities of PEG leaching from the MAPs exceed those of Gantrez^®^ S-97. This outcome is expected, as PEG molecules can form ester bonds only via their two terminal alcohol groups, whereas Gantrez^®^ S-97 has two acid groups per monomer, allowing for more bonding opportunities. Such stress-testing aligns with regulatory expectations for extractables analysis, which often involves the use of aggressive solvents and elevated temperatures to simulate worst-case scenarios and ensure the safety of materials intended for prolonged skin contact.

### MALDI-TOF analysis

**Fig. 8:**
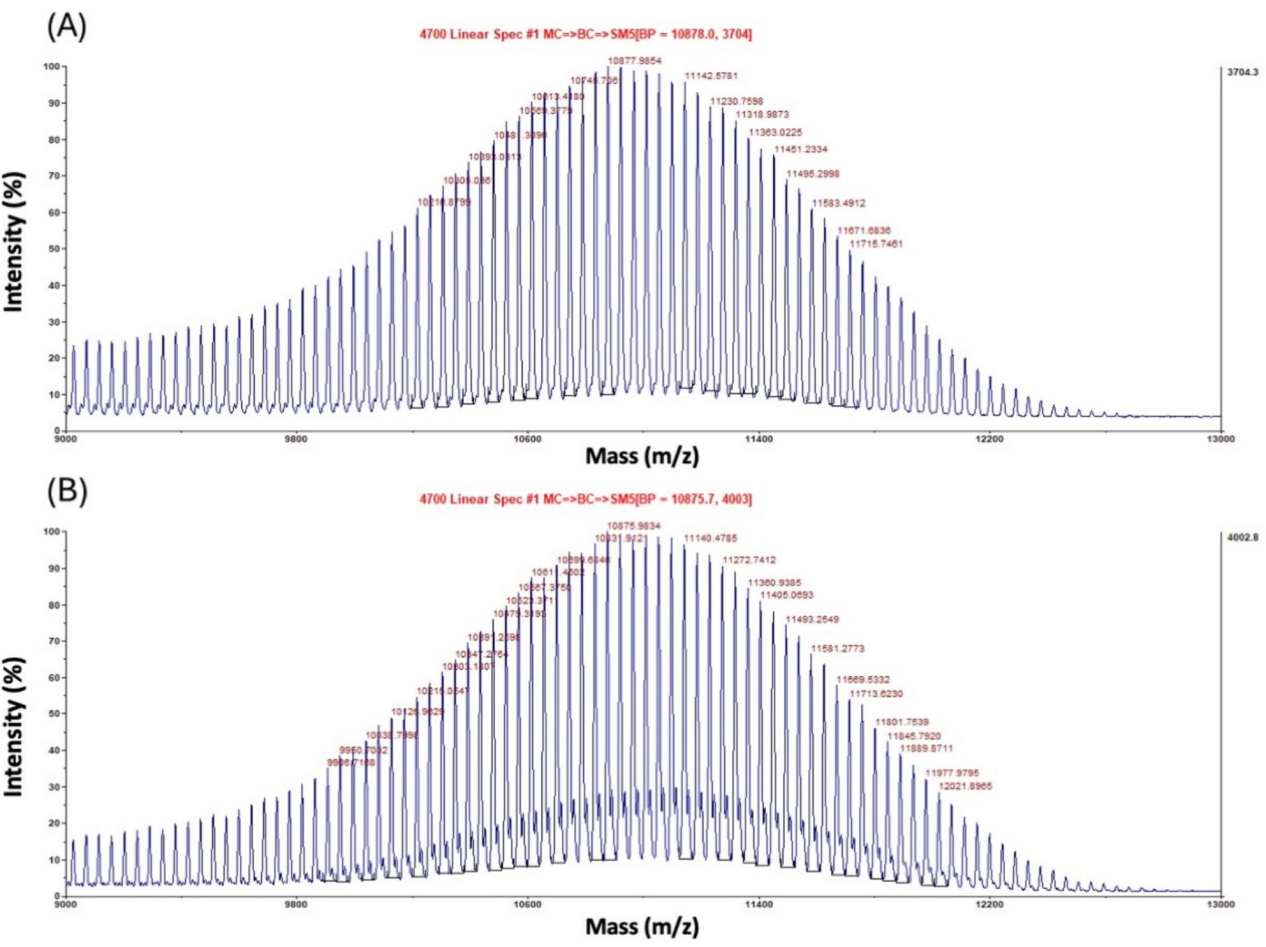
MALDI-TOF MS spectra obtained from (**A**) release samples of hydrogel-forming MAPs (treated at room temperature for 24 h) in contact with water as the release media. (**B**) PEG 10,000 at 1 mg/mL (w/v) (control)

MALDI-TOF MS analysis was able to identify the PEG release after the 24-h experiment from hydrogel-forming MAPs (Fig. 8A). This analysis was able to confirm that a compound with a molecular weight of 10,878.0 Da was being released from the hydrogel- forming MAPs. The profile can be attributed to PEG 10,000, as the control sample (1 mg/mL of PEG 10,000 in water, w/v) (Fig. 8B) shows the exact mass of 10,875.7 Da. However, Gantrez^®^ S-97 (m/z 1,500,000 Da or 1,500 kDa), could not be detected by this instrument, as the maximum scanning mass is up to m/z 200,000 Da (200 kDa).

## Discussion

In this work, protocol one was used to determine the leachable compounds from “super-swelling” hydrogel-forming MAPs prepared from Gantrez^®^ S-97, PEG and Na_2_CO_3_ following equilibration in H_2_O at a temperature of 37℃ ± 1 °C for 24 h [3]. FTIR, LC–MS and NMR analyses were used to confirm the presence of PEG 10,000 in protocol one samples. This was then corroborated with findings from HPLC-ELSD analysis, which revealed the concentration of PEG 10,000 in these samples to be 0.86 ± 0.11 mg/mL. Based on the starting mass of the hydrogel-forming MAPs used and the volume of H_2_O remaining at 24 h, this equated to 10.4 ± 2.0% (2.8 ± 0.5 mg) of total PEG 10,000 content leached from hydrogel-forming MAPs after 24 h (Table 1). As detailed previously, cross-linking of PEG 10,000 and Gantrez^®^ S-97 via esterification is carried out to create an insoluble, yet swellable, polymeric matrix *i.e.,* a hydrogel. Whilst the cross-linking process employed here is effective, it is not complete. As a result, a small proportion of polymer remains uncross-linked and, therefore, free to leach from the hydrogel. In this instance, uncross-linked PEG 10,000 that had leached from the hydrogel was identified and, subsequently, quantified. Although Gantrez^®^ S-97 is the main polymeric constituent of the hydrogel small amounts of Gantrez^®^ S-97 were extracted. Less than 2% of the initial amount of Gantrez^®^S-97 was extracted. As mentioned earlier, it is not surprising that higher amounts of PEG are extracted than Gantrez^®^ S-97 as the later has more functional groups available for reaction.

Analysis of samples from protocol two provided insight into the compounds present in H_2_O that had been absorbed by a hydrogel-forming MAP and, subsequently, extracted from the MAP using centrifugation. FTIR and NMR analyses indicated that polymeric compounds, similar in structure to PEG 10,000, were present within these samples at higher concentrations than protocol one samples. Once again, LC–MS was used to confirm the presence of PEG 10,000 followed by HPLC-ELSD which indicated that the concentration of PEG 10,000 in these samples was 2.84 ± 1.21 mg/mL. Based on the starting mass of the hydrogel-forming MAPs used here and the volume of H_2_O extracted after centrifugation, this equated to 14.8 ± 4.6% (4.0 ± 1.4 mg) of total PEG 10,000 content extracted from hydrogel-forming MAPs after 24 h. It should be noted that the MW cut-off of the centrifugal filters used to extract H_2_O from swollen MAPs was 100 kDa which, therefore, would eliminate Gantrez^®^ S-97 (MW = 1,500–2,000 kDa) from the obtained filtrate.

Protocol three sought to expose hydrogel-forming MAPs to a high level of stress to identify additional compounds outside of those likely to be encountered during appropriate MAP use, thereby, expanding the obtained safety profile. This was achieved by allowing hydrogel-forming MAPs to swell to equilibrium in the solvent DMSO at a temperature of 70℃ ± 1℃ and then analysing un-absorbed solvent. After 24 h, visible breakdown of the swollen hydrogel had occurred with reduced structural integrity apparent. Under these conditions, 5.3 ± 2.6 mg of PEG 10,000 had been extracted, which equated to 19.5 ± 8.6% of total PEG 10,000 content. The increased PEG 10,000 content observed here is likely due to the presence of DMSO. This organic solvent is a better solvent for PEG than water, as reported previously [40]. Interestingly, NMR analysis identified the presence of multiple compounds that were similar, although not identical, in structure to Gantrez^®^ S-97. Furthermore, the presence of a peak at 206.99 ppm in ^13^C-spectra indicated that a compound containing an anhydride group had been extracted. It is likely that this compound was the anhydride form of Gantrez^®^ S-97, which has previously been reported as a minor side product of the cross-linking process wherein adjacent acid groups on a Gantrez^®^ S-97 molecule react with each other rather than the terminal hydroxyl groups on PEG 10,000 [41, 42]. In such instances, successful cross-linking does not occur, and the anhydride form of Gantrez^®^ S-97 can be extracted from the hydrogel upon sufficient breakdown of its overall structure by DMSO and heated at 70 °C. NMR analysis of these samples was carried out using deuterated DMSO, the peak at 3.35 ppm in ^1^H-spectra confirmed the presence of residual H_2_O in hydrogel-forming MAPs in their dried xerogel state. Finally, H-NMR analysis of the extracted samples was used to calculate the amount of Gantrez^®^ S-97 extracted from the samples. The amount of Gantrez^®^S-97 extracted under DMSO is similar to the one obtained in water (Protocol 1) and therefore we can conclude that even if more PEG was extracted, the amount of unbound Gantrez^®^ S-97 was equivalent to the values obtained for Protocol 1.

In a similar manner to protocol three, protocol four sought to expose hydrogel-forming MAPs to a high level of stress by allowing them to equilibrate in H_2_O at an elevated temperature (90℃ ± 1℃). Once again PEG 10,000 was the most abundant extractable compound with 8.8 ± 1.9 mg (32.9 ± 6.1% of total PEG 10,000 content) extracted after 24 h. Of all the protocols tested, samples from protocol four contained the greatest amount of extracted PEG 10,000. This may be attributed to the elevated temperature of the surrounding solvent which corresponded with increased hydrogel swelling and elevated outward diffusion of PEG 10,000 from the hydrogel matrix. Moreover, higher temperatures will lead to faster ester hydrolysis as reported previously [36]. As with protocol three samples, compounds that were similar, although not identical, in structure to Gantrez^®^ S-97 were found in samples from protocol four. The anhydride form of Gantrez^®^ S-97 was not identified in these samples; therefore, it was hypothesised that further degradation of Gantrez^®^ S-97 and the anhydride form of Gantrez^®^ S-97 had occurred here compared to protocol three samples. Alternative NMR analysis suggested ester hydrolysis as slightly higher amounts of Gantrez^®^ S-97 were extracted at higher temperatures.

The findings from this study demonstrate that hydrogel-forming MAPs exhibit minimal leaching of PEG 10,000 and negligible extraction of Gantrez^®^ S-97 under physiological conditions. According to material safety data, the acute toxicity of Gantrez^®^ S-97 and PEG occurs in rats at doses exceeding 6,450 mg/kg and 2,000 mg/kg via oral administration, respectively [43, 44]. The levels of Gantrez^®^ S-97 and PEG leached and extracted in this study remain well within the established safety range, reinforcing the biocompatibility of the MAP formulation for transdermal drug delivery. Clinically, this type of MAP is particularly suitable for managing chronic conditions requiring repeated dosing. These findings highlight the potential of hydrogel-forming MAPs to overcome the limitations of conventional drug delivery methods. However, further clinical studies are needed to validate these preclinical findings regarding leachables in human subjects, supporting regulatory approval and broader adoption of this innovative technology across various therapeutic applications.

## Conclusion

This study evaluated the leachable compounds from hydrogel-forming MAPs composed of Gantrez^®^ S-97, PEG, and sodium bicarbonate under various conditions, providing critical insights into their safety and clinical potential. Under physiological conditions (water at 37 °C), only a small proportion of PEG 10,000 (10.4 ± 2.0%) and negligible amounts of Gantrez^®^ S-97 (< 2%) were leached, confirming the stability and safety of the cross-linked hydrogel for its intended use. Stress tests under extreme conditions, including exposure to DMSO and elevated temperatures, revealed increased extraction of PEG 10,000 and minor degradation of Gantrez^®^ S-97 via anhydride formation, further demonstrating the MAPs’ robustness and safety profile. These findings support their suitability for chronic therapeutic applications requiring repeated and sustained drug administration. Future research should focus on human clinical studies to validate these preclinical findings, facilitate regulatory approval, and promote the broader adoption of hydrogel-forming MAPs in advanced drug delivery systems.

## Data Availability

The data is available from the corresponding author upon request.
